# Metal chelation reduces skin epithelial inflammation and rescues epithelial cells from toxicity due to thermal injury in a rat model

**DOI:** 10.1093/burnst/tkaa024

**Published:** 2020-10-02

**Authors:** Amina El Ayadi, Cheng Z Wang, Min Zhang, Michael Wetzel, Anesh Prasai, Celeste C Finnerty, Perenlei Enkhbaatar, David N Herndon, Naseem H Ansari

**Affiliations:** Department of Surgery, University of Texas Medical Branch, Galveston, TX 77555, USA; Department of Biochemistry and Molecular Biology, University of Texas Medical Branch, Galveston, TX 77555, USA; Department of Biochemistry and Molecular Biology, University of Texas Medical Branch, Galveston, TX 77555, USA; Department of Surgery, University of Texas Medical Branch, Galveston, TX 77555, USA; Department of Surgery, University of Texas Medical Branch, Galveston, TX 77555, USA; Department of Surgery, University of Texas Medical Branch, Galveston, TX 77555, USA; Department of Anesthesiology, 301 University Blvd., University of Texas Medical Branch, Galveston, TX 77555, USA; Department of Surgery, University of Texas Medical Branch, Galveston, TX 77555, USA; Department of Biochemistry and Molecular Biology, University of Texas Medical Branch, Galveston, TX 77555, USA

**Keywords:** Burn progression, Cytokines, Oxidative stress, Cell death, Wound healing, Inflammation, Metal chelation, skin

## Abstract

**Background:**

One of the most pervasive complications of burn injury is wound progression, characterized by continuous tissue destruction in untreated wounds, which leads to wound infection, inflammation, oxidative stress and excessive scar formation. We determined whether additional tissue destruction could be attenuated with Livionex formulation (LF) lotion, which contains a metal-chelating agent and reduces inflammation in burn wounds.

**Methods:**

We subjected male Sprague Dawley rats to a 2% total body surface area (TBSA) burn using a brass comb model and topically applied LF lotion (containing ethylenediaminetetraacetic acid and methyl sulfonyl methane) to the affected area every 8 hours over 3 days. Inflammatory cytokine levels, cell apoptosis and wound healing were compared in LF lotion-treated and untreated rats. Statistical analysis was performed using a one-way analysis of variance in conjunction with Tukey’s post-hoc test.

**Results:**

Serum inflammatory cytokines were not detectable after 3 days, suggesting that small burn wounds induce only an immediate, localized inflammatory response. Microscopy revealed that LF lotion improved burn site pathology. Deoxynucleotidyl transferase biotin-d-UTP nick-end labeling staining showed reduced cell death in the LF-treated samples. LF lotion prevented the spread of tissue damage, as seen by increased amounts of Ki-67-positive nuclei in the adjacent epidermis and hair follicles. Tumor necrosis factor-alpha, interleukin-6 and inducible nitric oxide synthase levels in LF-treated skin sections from burned rats were comparable to the levels observed in unburned control sections, indicating that LF lotion reduces inflammation in and around the burn site.

**Conclusions:**

These results establish LF lotion as a therapeutic agent for reducing inflammatory stress, cell death and tissue destruction when applied immediately after a burn injury. Further studies of LF lotion on large TBSA burns will determine its efficacy as an emergency treatment for reducing long-term morbidity and scarring.

Highlights

- This atudy examine a novel strategy to ameliorate progression of full thickness burn.
- The mechanism relies on the use of a metal chelator to control oxidative damage, inflammation and resulting tissue injury.
- Involvement of toxic lipid aldehydes, such as 4-hydroxy nonenal (HNE), suggestive in burn injury.
- Impaired HNE detoxification contributes to inflammation, apoptosis and other downstream events causing collagen destruction, cellular damage, cell necrosis leading to burn injury.
- Possible intervention of burn injury by topical application of metal chelator.

## Background

Traumatic burns cause significant psychosocial and economic burden to patients, their families and society. Worldwide, burn injuries lead to about 330,000 deaths yearly [1]. In the US, more than 2 million individuals sustain burns annually [2], with $18 billion spent on the specialized care of burn patients [3].

Burn wound progression refers to tissue destruction in untreated burn wounds, in the absence of infection, which continues to develop after the reduction of the initial burn insult [4]. Thus, a partial-thickness second-degree burn (especially a deep second-degree burn) becomes a full-thickness burn (third-degree) as the burn wound progresses [4–6]. The injury progression itself may change individual treatment paradigms, including increasing the need for burn wound excision or subsequent skin grafting. Accordingly, the likelihood of severe scarring, contracture, local discomfort and/or even disability may increase. Although estimates of the percentage of second-degree burn wounds converting to third-degree burn wounds are not known, the wound progression may aggravate the initial injury. Systemic inflammation and/or the oxidative stress response may be exacerbated during burn progression, leading to the deterioration of local and systemic homeostasis and immunity [7]. These sequelae significantly increase the risk of infection, the length of hospital stay and the cost of specialized care. It is therefore imperative to develop efficacious therapeutic interventions that will limit or block the burn wound progression.

Major efforts have been made to understand the mechanisms underlying burn wound progression and to seek topical and/or systemic therapeutic interventions [6, 8]. Proposed mechanisms underlying burn wound progression include acute thrombosis with a dermal and subcutaneous vascular blockade, reduction of local perfusion, oxidative stress [6, 8–10] and excessive inflammatory responses (as reviewed Schmauss *et al*. [11]). Accordingly, efforts to develop therapeutic interventions have targeted each of these mechanisms [12–14].

Previous studies by Tobalem *et al*. have also addressed the role of ischemia in burn wound conversion and treatment strategies to prevent the zone of stasis from becoming necrotic, including early treatment with erythropoietin, which is known to induce angiogenesis and prevent ischemia-induced cell death and inflammation [15, 16]. The same group has shown that early intervention (45 minutes post-burn) is more effective than late treatments (6 hours post-burn) in reducing burn wound progression [15] [17]. Severe burn is associated with increased levels of local and systemic inflammatory cytokines, such as interleukin-6 (IL-6) and tumor necrosis factor-alpha (TNF-α), or chemokines, such as interleukin-8 [17]. Inflammatory cytokines activate a cascade that leads to the generation of reactive oxygen species (ROS) [18–20], lipid peroxidation and cellular injury [21–23]. The polyunsaturated fatty acids localized to the plasma membrane are prone to lipid peroxidation and yield toxic lipid aldehydes [24, 25]. Lipid aldehyde production is also induced by trace metals like iron and copper [26, 27], although the mechanisms are not entirely understood [28]. We reported that the topical application of metal chelators, like ethylenediaminetetraacetic acid (EDTA), inhibits the oxidative and inflammatory insults in animal models of glaucoma [29], uveitis [30] and diabetic cataract [31].

Using a modified rat brass comb burn model, we recently tested our hypothesis that topical application of a lotion containing EDTA and methyl sulfonyl methane (MSM), Livionex formulation (LF) lotion, chelates the redox-active metals, blocks the formation of ROS and attenuates burn wound progression [32]. We reported that the application of LF lotion immediately post-burn significantly reduces the accumulation of reactive aldehydes and lipid peroxidation product 4-hydroxynonenal (malondialdehyde and acrolein) in the interspaces [32]. Morphologically, topical LF lotion application mildly reduced the histological characteristics of burn wound progression when compared to untreated burn wounds, including a depth of epithelial and endothelial cell necrosis, collagen denaturation, vessel blockade, and skeletal muscle damage [32]. The LF lotion-treated interspaces exhibit moderate morphological improvements, including reduced destruction and discoloration of the skeletal muscle, a reduced vascular expansion and congestion of the capillary loops and sub-papillary plexus, fewer inflammatory cells around expanded and clogged arteries in the hypodermis and an increase in the vertical length of survived interspaces [32].

This report investigates the effects of LF lotion on local and systemic inflammatory responses and epithelial cell death and proliferation in the burn sites and the non-burned interspaces 72 hours after burn. We measured the concentrations of plasma cytokines and chemokines to monitor systemic inflammation. Deoxynucleotidyl transferase biotin-d-UTP nick-end labeling (TUNEL) was used as an indicator of cell death. We examined whether burn progression impairs cell proliferation and if the application of LF lotion might reverse this effect. Local tissue inflammatory response was assessed via immunohistochemistry. We found that immediate topical application of LF lotion not only reduced positive TUNEL labeling and increased Ki-67 immunolabeling in epithelial structures of the burn site, but also prevented cell death and enabled cell proliferation in the unburned interspaces. This suggests that LF lotion efficiently reduces burn wound progression.

## Methods

### LF lotion and brass comb

As described previously [32], LF lotion (a proprietary lotion containing EDTA and MSM), and a control lotion/vehicle (a proprietary lotion without EDTA and MSM) was provided by Livionex Inc. (USA). We used a modification of the brass comb described in the Regas and Ehrlich model [33] with three 10-mm teeth separated by two 10-mm notches, instead of 5 mm, as used by Regas and Ehrich, to increase the surface of the interspaces and follow burn wound progression without interfering with the next burn area. The brass comb yielded three 10×19 mm rectangular contact burn sites, representing the zone of coagulation, separated by two 10×19 mm rectangles of the unburned interspace, which modeled the zone of stasis or ischemia.

### Animals

Male Sprague Dawley rats weighing ~350 g (Charles River Laboratories International, USA) were housed individually in a temperature- and humidity-controlled environment with a 12-hour light–dark cycle. During the acclimation period, the rats received standard diet and water *ad libitum* for at least a week before the experiment. All experiments complied with the National Institutes of Health (NIH) ‘Guide for the Care and Use of Laboratory Animals’ and were approved by the Institutional Animal Care and Use Committee of the University of Texas Medical Branch at Galveston.

### Experimental protocol

Experimental details are described elsewhere [32]. Briefly, the animals were anesthetized using 90 mg/kg of ketamine (10%) and 10 mg/kg of xylazine (2%) (both intraperitoneal). The dorsum of each animal was then shaved. A 19×50 mm rectangle was marked parallel to, and approximately 10 mm distal to, either side of the dorsal middle line between the caudal end of the scapula and rostral end of the ilium, resulting in two burn areas, one on each side of the animal. Rats were randomly assigned to four treatment groups (*n* = 5 each): sham control, burn alone, burn plus vehicle and burn plus LF lotion. Sham control animals were treated the same way as the burn animals, except for the burn. The burn was induced by applying a brass comb (which was preheated in boiling water (100°C) for 3 minutes) on each pre-marked rectangle with no additional pressure for 30 seconds. Within 5 minutes post-burn, we applied LF lotion (~1 mL) or vehicle, covering the entire burn region inclusive of burn sites as well as unburned interspaces. LF lotion application was repeated every 8 hours for 3 days. After the burn procedure, the animals were placed on a heating pad with oxygen provided until they recovered fully from the anesthesia. The animals were given buprenorphine (0.01 mg/kg, intraperitoneal) for the first 24 hours after the burn to relieve pain and discomfort. The animals were euthanized by decapitation 72 hours post-burn and blood and skin samples collected.

### Determination of plasma cytokines and chemokines

We determined plasma levels of cytokines and chemokines with the use of a MILLIPLEX MAG rat cytokine/chemokine magnetic bead enzyme-linked immunosorbent assay (ELISA) kit, along with Luminex xMAP detection, according to the manufacturer’s instructions (Biorad Laboratories, Hercules, CA). We also measured the levels of individual cytokines using ELISA kits from R&D systems (Minneapolis, MN). Data were presented as picograms per milliliter.

### Sample harvesting

**Figure 1:**
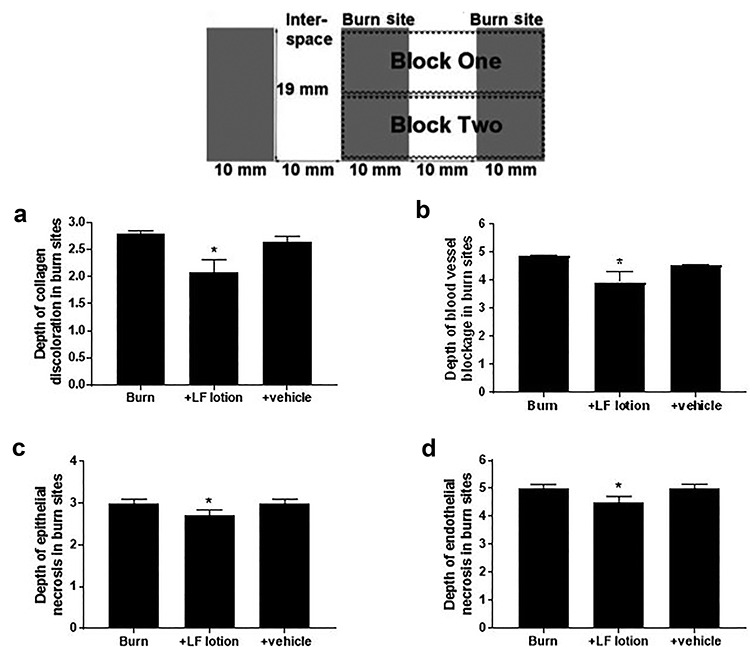
Microscopic scoring of the depth of (**a**) collagen discoloration, (**b**) blood vessel blockage, (**c**) epithelial cell necrosis and (**d**) endothelial cell death. ^*^p < 0.05 vs. burn alone or burn plus vehicle lotion. The inset shows each sampling block that encompasses 2 burn sites and 1 inter-space from each burn area (Block1 and Block 2). *LF* Livionex formulation

As elucidated in the inset of Figure 1 and previously published [32], 2 skin tissue blocks were harvested from each wound immediately after sacrifice and fixed in 10% neutral buffered formalin before embedding in paraffin. Tissue blocks were cut into 5-μm sections and stored at −20°C until processing for histochemistry, TUNEL labeling or immunohistochemistry.

### Microscopic analysis of the burn site pathology

Skin sections were deparaffinized, rehydrated and stained with hematoxylin–eosin (Hematoxylin Stain Harris Formulation and Eosin Y; StatLab, USA) and Masson’s trichrome (Sigma-Aldrich Corporation, USA). Burn wound depth progression (vertical progression) was determined by measuring the depth of collagen discoloration in Masson trichrome-stained sections and scored as described in Table 1. Vertical burn wound progression was defined by measuring the microscopic depth of blood vessel blockage and necrosis of epithelial and endothelial cells [34]. The depth of these lesions was scored as detailed in Table 2. The scoring was performed in the middle of both burn sites for each segment, resulting in eight scores from each animal. Each animal’s scores were then averaged to determine one value for that animal. Assessments and scoring were performed by two observers blinded to the treatment. The score was accepted when the two observers agreed on the scoring.

### TUNEL labeling

Formalin-fixed paraffin-embedded skin sections were deparaffinized, rehydrated and then TUNEL stained as described before [35], with modification. The sections were incubated in 0.85% NaCl and 0.01 M phosphate buffered saline (PBS) for 5 minutes each, fixed in 4% paraformaldehyde in PBS for 15 minutes and then washed with PBS. The sections were then incubated with proteinase K (20 μg/ml) for 10 minutes, washed with PBS, blocked with avidin/biotin solutions (Invitrogen, USA) for 15 minutes each and incubated in a TUNEL reaction buffer (30 mM Tris-HCl, pH 7.2; 140 mM sodium cacodylate; and 1 mM CoCl_2_). Subsequently, the sections were incubated in a humidified chamber for 90 minutes at 37°C with TUNEL reaction mixture-containing biotin-16-dUTP (10 nmol/ml) (Roche, USA) and terminal deoxynucleotide transferase (TdT) (200 U/ml) (Roche). Negative controls were incubated without TdT. Sections were then washed in PBS, incubated with ABC reagents (Vectastain ABC Elite Kit; Vector Laboratories, USA) for 60 minutes and stained with 3, 3 –diaminobenzidine (DAB) substrate (Dako, USA).

**Table 1:** Measurements and microscopic scoring of collagen discoloration in burn sites

Score	Depth of collagen discoloration
0	No discoloration
1	Discoloration at the top half of dermis
2	Discoloration extending below the hair follicles
3	Discoloration through 25% of the hypodermis
4	Discoloration through 50% of the hypodermis
5	Discoloration in the whole hypodermis

### Immunohistochemistry

Paraffin-embedded skin sections were deparaffinized and rehydrated. After antigen retrieval with citrate buffer the sections were washed with PBS. Sections were treated with 3% hydrogen peroxide/methanol to block endogenous peroxidases before washing and incubating in a blocking solution (3% normal goat serum/2% total body surface area (TBSA)/0.1% cold fish skin gelatin/0.1% Triton X-100/0.05% Tween 20/0.05% sodium azide in 0.01 M PBS). The sections were then blocked with avidin/biotin and incubated with the primary antibody. After washes and incubation with secondary biotinylated antibodies, the sections were processed with ABC reagents and developed with DAB substrate. All sections were counterstained with 0.5% methyl green. The optimal concentration of primary antibody and negative control staining was predetermined for each immunohistochemistry (IHC) experiment. For each negative control, the primary antibody incubation was omitted. The following primary antibodies were used in this study: rabbit polyclonal anti-TNF-α (#NBP1–19532) and rabbit anti-Ki-67 (#NB500–170) (both from Novus Biologicals, USA); and rabbit polyclonal anti-inducible nitric oxide synthase (iNOS) (#ab15323) and rabbit polyclonal anti-IL-6 (#ab6672) (both from Abcam, USA).

**Table 2:** Measurements and microscopic scoring of the pathology of interest in burn sites

Score	Depth of vessel blockade and cell necrosis (or lesion)
0	No lesion
1	Lesion limited at or above the epidermis
2	Lesion in the dermis but above the bottom of hair follicles
3	Lesion down to the hypodermis but above the skeletal muscle
4	Lesion down to skeletal muscle
5	Lesion down below the skeletal muscle

### Image visualization, acquisition and analysis

All TUNEL-labeled and immunohistochemical-stained skin sections were imaged with an Olympus BX53 digital microscope equipped with the CellSense Program CellSense program ( Olympus, USA). Analysis of TUNEL-labeled images was carried out using ImageJ software from the NIH to automatically measure the mean intensity of DAB staining in the structures of interest, such as the epidermis and hair follicles. The average value of images taken from 8 burn sites from each animal was used to represent that animal.

**Figure 2.**
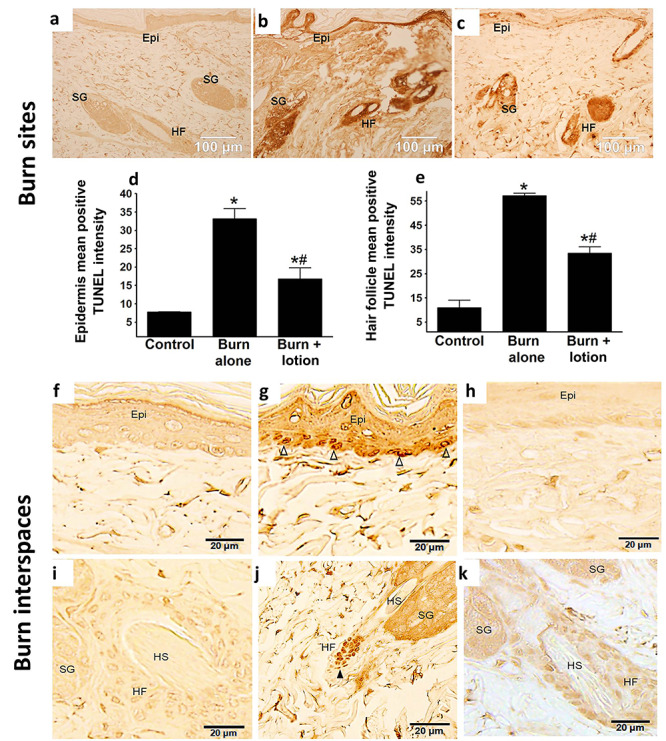
LF lotion reduces the burn-induced apoptosis in the burn sites and interspaces. **(a–c)** Representative micrographs of deoxynucleotidyl transferase biotin-d-UTP nick-end labeling (TUNEL) staining in the burn site showing extensive labeling in the epidermis, hair follicles and sebaceous glands in burn-alone animals but significantly less staining in controls and Livionex formulation (LF) lotion-treated animals. **(a)** Control, with neither burn nor any lotion; **(b)** burn site 72 hours post-burn alone; **(c)** burn site 72 hours post-burn plus topical application of LF lotion every 8 hours starting 5 minutes after the burn; **(d)** mean positive TUNEL intensity of epidermis; **(e)** mean positive TUNEL intensity of hair follicles. TUNEL staining in interspace epidermis **(f–h)** and hair follicle **(i–k)**, showing strong TUNEL-positive labeling of individual epithelial nuclei scattered over the basement of the epidermis **(g)** or focused on the hair follicles **(j)** of burn-only animals. The control sections (panels **(f)** or **(i)**) and LF lotion-treated sections (panels **(h)** or **(k)**) showed very few positive TUNEL-stained individual epithelial nuclei in the epidermis (panels **(f)** and **(h)**) and hair follicles (panels **(i)** and **(k)**). **(f)** and **(i)**, sham control; **(g)** and **(j)**, 72 hours post-burn alone; **(h)** and **(k)**, 72 hours post-burn and topical application of LF lotion every 8 hours starting 5 minutes right after the injury. ^*^p < 0.05, vs. control, #*p* < 0.05 vs. burn alone. Scale bar = 100 μm in **(a–c)***,* 20 μm in **(f–k)**. Open triangles Δ indicate basal cells with TUNEL-positive staining, black triangles ▲ point to the hair follicle epithelial cells with TUNEL-positive staining. *Ep*, epidermis, *SG*, sebaceous gland, *HF* hair follicles, *HS* hair shaft

### Statistical analysis

Data are presented as the mean ± standard deviation. Statistical analysis was performed using a one-way analysis of variance in conjunction with Tukey’s post-hoc test (GraphPad, USA). Differences were considered significant at *p* < 0.05.

## Results

### Burn size and plasma concentrations of cytokines and chemokines

TBSA was estimated using Meeh’s formula (TBSA = *kW*^2/3^) with a *k* value of 9.83 [36]. TBSA values were 558 ± 15 cm^2^ in control animals, 538 ± 9 cm^2^ in the burn-alone group and 564 ± 7 cm^2^ in the burn-plus-LF lotion rats, showing no significant differences among the three groups (*p* > 0.05). No significant change for burn size (percent of TBSA burned) was seen between the 2 burn groups: 2.12% ± 0.02 in the burn-alone group and 2.02% ± 0.02 in the burn-plus-LF lotion group (*p* > 0.05).

We measured plasma concentrations of a total of 23 cytokines and chemokines (data not shown). Only three cytokines, IL-12p70, GRO-KC and Leptin were detectable but showed no significant differences among the three groups. The remaining 20 cytokines/chemokines were not detectable. They included: IL-1β, IL-1, IL-2, IL-4, IL-5, IL-6, IL-10, IL-13, IL-17, IL-18, IP-10, MCP-1, MIP-1a, RANTES, Eotaxin, G-CSF, GM-CSF, IFNγ, TNF-α and VEGF. It appears that a burn size as small as 2% TBSA did not induce a significant systemic inflammatory response. However, since these results were obtained 72 hours post-burn, they only suggest that there is no long-lasting systemic inflammatory response and an acute systemic inflammatory response, as reported by others [17, 37], cannot be excluded.

### Microscopic analysis to determine the effect of burn on skin histology

As presented in Figure 1 and Tables 1 and 2, and as reported earlier [32], the untreated burn sites displayed various pathological microscopic changes post-burn that were significantly improved in the LF-treated burn sites. Notably, the vehicle-treated burn sites did not show any significant improvement of the burn-induced pathology. Therefore, we excluded the specimens from the vehicle-treated group in all subsequent studies and limited the data analysis to the following groups: control, burn alone and burn-plus-LF lotion.

### TUNEL labeling in epithelial structures

In tissue sections from control animals there was no positive TUNEL labeling in individual epithelial cell nuclei in either the epidermis or hair follicles (Figure 2a). In burn-alone animals, extensive positive TUNEL staining was observed in the epidermis, hair follicles and sebaceous glands in the burn sites (Figure 2b) compared to the surrounding dermal areas in the same sections. The staining intensity was also higher in these animals when compared to similar structures in control animals without burn or lotion application (Figure 2a). In the burn sites of the LF lotion-treated animals (Figure 2c), TUNEL labeling was reduced in the hair follicles, epidermis and sebaceous glands, when compared to the burn-alone animals (Figure 2b). Quantification of the TUNEL staining intensity in burn sites further confirmed that LF lotion did not completely eradicate TUNEL staining but significantly reduced the intensity of positive TUNEL staining in the epidermis (Figure 2d), hair follicles (Figure 2e) and sebaceous glands (data not shown) in the burn sites.

In the interspaces of TUNEL-stained burn sections (Figure 2f–k), epithelial structures did not show a smear-like staining compared to the controls (Figure 2f, i). instead, a strong TUNEL staining of individual epithelial cell nuclei in the epidermis and hair follicles was recorded (Figure 2g, j). Under high magnification, individual nuclei with strong positive TUNEL staining were seen scattered and lined up above the basement membrane, and most of the TUNEL-positive cells were located in the basal layer (Figure 2g). Unlike the epidermis, the hair follicles showed not only a more extensive DAB staining intensity (compared to the control) but also tiny focal patches of individual nuclei with positive TUNEL labeling (Figure 2j). The small patches appear as clusters of individual epithelial cell nuclei with strong TUNEL staining at the base of each hair follicle. TUNEL staining of sebaceous glands in burn-alone sections was more intense and no epithelial nuclei showed strong positive TUNEL labeling (Figure 2j). TUNEL staining intensity in the epidermis, hair follicles and sebaceous glands of LF lotion-treated animals (Figure 2h, k) was comparable to that seen in sections from control animals (Figure 2f, i). No individual epithelial nuclei with strong TUNEL labeling were noted, demonstrating that early and continuous application of LF lotion prevented epithelial cell death in the epidermis and hair follicles of the interspaces. The epithelial cell death in the interspaces might be a direct result of burn wound progression over the 72 hours. Application of LF lotion prevented burn wound progression to nearby interspaces in this model.

**Figure 3.**
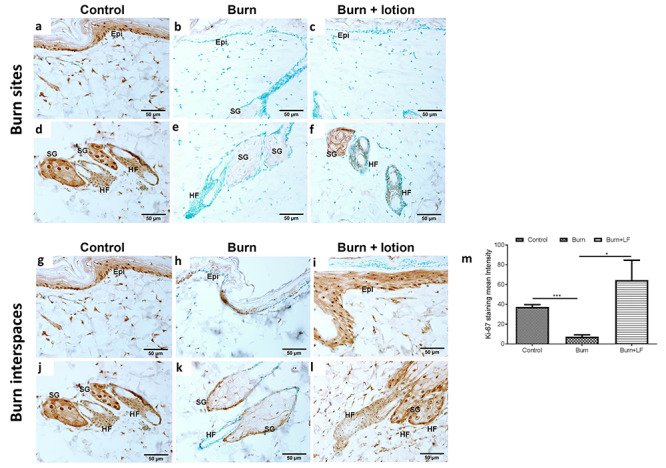
Ki-67 immunostaining in the burn sites and interspaces. **(a–c)** Representative micrographs of Ki-67 immunostaining in the burn site epidermis and **(d–f)** the hair follicles, showing the absence of Ki-67 immunoreactivity in the epidermis of both **(b)** burn-alone and **(c)** LF lotion-treated animals. The hair follicles, however, showed higher immunoreactivity to Ki-67 in **(f)** the Livionex formulation (LF) lotion-treated sections compared to **(e)** the burn-alone animals. **(g–l)** In the interspace, Ki-67 immunostaining in **(g–i)** the epidermis and **(j–l)** hair follicles showed a dramatic decrease in **(h)** the epithelial cells of the epidermis, sebaceous gland and **(k)** hair follicles of burn-alone animals. In the LF lotion-treated animals (panels **(i)** and **(l)**), both **(i)** epidermis and **(l)** hair follicles showed a similar number of Ki-67-positive cells to that in the control animals (panels **(g)** and **(j)**). The quantification of Ki-67 staining intensity in panels **(g)**, **(h)** and **(i)** is shown in panel **(m)**. Panels **(a)**, **(d)**, **(g)** and **(j)** depict controls, with no burn or lotion; (**b**), (**e**) and (**h**), (**k**), burn site 72 hours after 30 seconds burn alone; (**c**), (**f**) and (**i**), (**l**), burn site 72 hours after 30 seconds burn plus topical application of LF lotion every 8 hours started 5 minutes after the injury;. Scale bar = 50 μm. Data are mean ± SEM. ^*^p < 0.05, ^*^^*^^*^p < 0.001. *Epi* epidermis, *SG* sebaceous gland, *HF* hair follicles

**Figure 4.**
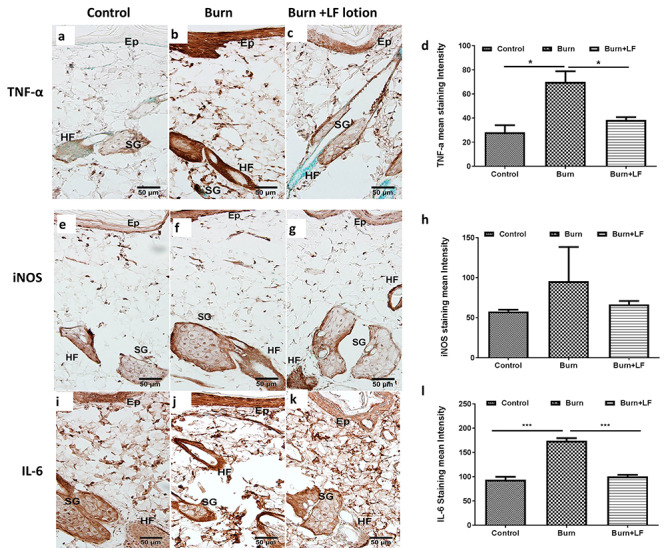
Representative immunohistochemistry (IHC) microphotographs counterstained with methyl green showing **(a–c)** positive immunostaining of tumor necrosis factor-alpha (TNF-α), **(e–g)** inducible nitric oxide synthase (iNOS) and **(i–k)** interleukin-6 (IL-6) in the interspaces of controls (panels **(a)**, **(e)** and **(i)**), burn-alone animals (panels **(b)**, **(f)** and **(j)**), and burn plus Livionex formulation (LF) lotion-treated animals (panels **(c)**, **(g)** and **(k)**). Samples were collected 72 hours post-burn. We applied LF lotion every 8 hours starting 5 minutes post-burn. Panels **(d)**, **(h)** and **(l)** show the respective quantification of the staining intensity for TNF-α, iNOS and IL-6. Scale bar = 50 μm. Data are mean ± standard error of the mean. ^*^p < 0.05, ^*^^*^^*^p < 0.001. *Epi* epidermis, *HF* hair follicles, *SG* sebaceous gland

### Epithelial Ki-67 expression

Ki-67 expression was observed in epithelial cell nuclei of the epidermis (Figure 3a), sebaceous glands and hair follicles (Figure 3d) of control animals. In burned animals, Ki-67 expression was not observed in individual cell nuclei of burn sites (Figure 3b, e), although light labeling was noted in the cytoplasm of sebaceous glands (Figure 3e). In the burn sites of the LF lotion-treated animals, the epidermis (Figure 3c) showed similar Ki-67 immunoreactivity to the burn-only animals (Figure 3b). However, Ki-67 immunoreactivity was significantly increased in the hair follicles of LF-treated animals (Figure 3f) compared to burn-only animals (Figure 3e). These data suggest that burn impairs the capability of cells around the epithelial region to proliferate and that LF lotion reverses this effect.

In the interspaces of burn-only animals, few Ki-67-positive nuclei were observed in the epidermis (Figure 3h) and sebaceous glands (Figure 3k), but no Ki-67 was seen in the hair follicles (Figure 3k), indicating that burn wound progression may impair epithelial cell proliferation in the epidermis (Figure 3h) and sebaceous glands (Figure 3k) and completely eradicate cell proliferation in the hair follicles (Figure 3k). When matched to controls (Figure 3g, j), LF lotion-treated interspaces presented comparable or even greater numbers of Ki-67-positive nuclei in all epithelial structures including the epidermis (Figure 3i), sebaceous glands and hair follicles (Figure 3l). These data were further supported by the quantification of Ki-67 immunostaining intensity in the epidermis (Figure 3m). Therefore, topical administration of LF lotion starting in the immediate aftermath of a burn reduced the progression of the burn wound to nearby interspaces and rescued epithelial cell proliferation in this model.

### Local expression of TNF-α, iNOS and IL-6 by IHC

TNF-α immunoreactivity in control skin sections was minimal (Figure 4a). Untreated burn sites stained similarly to controls for TNF-α (image not shown). In contrast, TNF-α immunostaining in the sebaceous glands, epidermal cells and hair follicles within the untreated interspaces (Figure 4b) was markedly increased. The interspaces treated with LF lotion showed marked reduction of TNF-α staining in the cells of the hair follicles (Figure 4c). These observations were confirmed by quantification of TNF-α immunoreactivity in the interspaces (Figure 4d), suggesting a protective effect of LF lotion from the harmful effects of TNF-α overproduction.

In control sections, iNOS immunoreactivity showed light-to-moderate intensity in all epithelial and mesenchymal cells (Figure 4e). The middle of the untreated burn sites exhibited minimal staining in epithelial cells but intense staining in mesenchymal cells within the hair follicle and hypodermis (images not shown). The center of untreated interspaces, however, showed increased iNOS immunoreactivity in the epidermal epithelial cells (Figure 4f) when compared to controls (Figure 4e), while the mesenchymal cells stained similarly to controls. Quantification of DAB staining intensity showed a burn-induced increase in iNOS immunoreactivity that did not reach significance (Figure 4h). The LF lotion-treated interspaces demonstrated the same intensity of iNOS staining in the epidermal epithelial cells compared to controls (Figure 4g, e), indicating that topical LF lotion application blocked burn-induced iNOS production.

IL-6 immunoreactivity was moderate-to-intense in the epithelial (Figure 4i) and mesenchymal cells (image not shown) of control animals. The untreated burn sites also showed moderate IL-6 immunoreactivity in all cellular structures except the hair follicle cells (image not shown). The untreated interspaces (Figure 4j) exhibited slightly higher IL-6 immunostaining and the LF-treated interspaces revealed similar staining intensity compared to the controls (Figure 4k, i). These data were confirmed by the assessment of IL-6 immunostaining intensity using ImageJ (Figure 4l).

## Discussion

Burn wound progression may convert a mild injury to a severe one. An efficacious therapeutic intervention should attenuate or stop burn wound progression. Such a therapeutic strategy would be beneficial for the management and healing of various types of burn wounds, including superficial (first-degree), superficial and deep partial-thickness (second-degree) and full-thickness (third-degree) burn wounds. Preventing burn wound progression may decrease the risk of developing wound infections, shorten the recovery time, decrease the need for surgical interventions, such as wound excision and grafting, and reduce the risk of scar formation and wound contraction. Various treatment strategies were used to attenuate burn wound progression, including erythropoietin [15, 16], keratin hydrogels [38], mesenchymal stem cell injections [39, 40], hypothermia [41] and increased perfusion of burn wounds [42]. Many molecular mechanisms have been found to be involved in burn wound conversion, including apoptosis [43, 44], necrosis [6, 45], ischemia [12, 33, 45, 46], oxidative stress [10, 15], inflammation [6, 17, 41] and autophagy [44]. While various treatment strategies have been investigated to target specific or groups of mechanisms involved in burn progression (as reviewed by Schmauss *et al*. [11]), we sought to use the LF lotion containing a metal chelator to determine the mechanism by which metal chelation might prevent burn wound conversion.

In this study, application of the LF lotion containing the reactive metal chelator EDTA and the excipient MSM significantly reduced positive TUNEL staining in the epithelial structures of burn sites and blocked cell death in the epidermis of the interspaces. Additionally, LF lotion successfully reversed the effects of burn wound progression on cell proliferation in the interspaces. These data suggest that reducing the formation of ROS by chelating reactive metals prevented burn wound progression to the interspaces and reduced the vertical and horizontal progression of burn wounds. We have previously reported that early application of LF lotion attenuates burn wound progression by decreasing the accumulation of reactive lipid aldehydes and protecting aldehyde dehydrogenase isozymes [32]. Together, these results demonstrate the implication of oxidative stress in burns and the therapeutic effects of this novel lotion.

**Figure 5.**
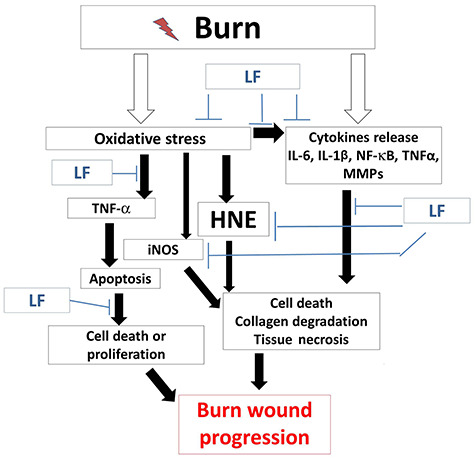
Schematic overview illustrating the mechanism underlying the various pathways targeted by Livionex formulation (LF) lotion to reduce burn wound progression. Since oxidative and inflammatory pathways are generally metal-catalysed, we propose that LF lotion will attenuate burn wound conversion by targeting various mechanisms activated after the burn to reduce the expression of the main downstream effectors. *IL-6* Interleukin-6, *IL-1β* interleukin-1 beta, *NF-κB* nuclear factor kappa-light-chain-enhancer of activated B cells, *MMPs* matrix metalloproteinases, *TNF-α* tumor necrosis factor-alpha, *HNE* 4-hydroxynonenal, *iNOS* inducible nitric oxide synthase

It is well documented that burns induce a significant increase in systemic inflammation [17, 37]. In the present study, systemic plasma cytokine and chemokine concentrations were not increased by 72 hours following the injury, which may be a result of the small burn size of this model (~2% of TBSA burned). This does not rule out a possible early increase in cytokine expression that has declined by 72 hours post-injury. In local wounds, however, the expression of IL-6, iNOS and TNF-α was still significantly increased at 72 hours as assessed by immunohistochemistry. Our results are in agreement with previous reports of both local and systemic increases of TNF-α levels post-burn [17]. More interestingly, the application of the LF lotion immediately post-injury significantly reduced the burn-induced TNF-α expression in the interspaces, indicating that reducing the formation of ROS attenuated the release of TNF-α in interspace epithelial structures, including the epidermis and hair follicles. These data are in accordance with the established relationship between the oxidative response and inflammatory mediators as elucidated in the study schematic overview (Figure 5) and reported in the literature [17–23]. While the burn-induced increased expression of iNOS did not reach significance in the time point examined in our study, LF lotion-treated animals maintained similar iNOS expression levels to control animals. These data agree with a previous report by Tobalem *et al*. showing that early erythropoietin treatment reduces burn wound progression independently of iNOS expression [15]. A similar conclusion may be drawn from our study. Early topical application of LF lotion also completely blocked epithelial cell death (as determined by positive TUNEL staining) and rescued the capability of epithelial cell proliferation.

TNF-α is well known to induce cell apoptosis through the TNF-α-induced signaling complex II pathway. Therefore, it is reasonable to conclude that TNF-α plays an important role in the pathogenesis of epithelial cell death in the interspaces of the epidermis and hair follicles in the current model (Figure 5). Although thermal injury-induced TNF-α elevation can initiate free radical production [47–49], the increase in ROS expression can also stimulate TNF-α generation [50]. An increase in ROS also enhances the production of toxic aldehydes that contribute to oxidation-induced injury [51–54], including burn injury [32]. The protective effect of LF lotion on burn sites is not as strong in the interspaces, suggesting that LF lotion prevents burn wound progression more horizontally than vertically. However, this may be due to the complexity of the contributors to burn wound progression. In addition to the heat-induced, direct, irreversible damage to skin components, the deeper layers of the skin, including epithelial cells (epidermal and hair follicle), endothelial cells of microvessels, dermal fibers (collagens) and mesenchymal cells, may also be damaged to an extent that may be reversible during a short period following the initial thermal injury (for example, within 1–2 hours) if there is no additional damage from complications such as infection. The pathology of burn wound progression is dynamic: it starts immediately after the initial injury and, depending on the severity, can last for several days thereafter. Locally, damaged cells or tissue components may release various bioactive substances including proteins, peptides or molecules, such as cytokines, chemokines, ROS species, vasoactive factors, blood cell activators, etc. Systemically, the initial burn triggers the response of multiple systems (immune, peripheral and central nervous and adrenal/cortex stress response systems), resulting in the release of a plethora of factors into the circulation. These factors are then carried to the wound where they act on the tissue responding to the injury. The bioactive substances from systemic and local sources form a complicated network in which there may be reciprocal causation. Each factor may be a product of other early response signaling mechanisms but then serve as an effector in later interactions. During this complicated process, reversible injuries to epithelial and endothelial cells, dermal fibers and mesenchymal cells become permanent, resulting in the cells being unable to recover, which may lead to additional wound excision and grafting procedures. Future approaches targeting multiple pathways simultaneously may be more efficacious in preventing vertical burn wound progression from the initial burn.

## Conclusions

In summary, the current study demonstrated that thermal injury induces a significant increase in cell death and expression of cytokines and inflammatory markers as examined 72 hours after injury. LF lotion acted as a therapeutic agent for reducing inflammatory stress, cell death and tissue destruction when applied immediately post-burn. Based on our previous and current studies, it appears that early iron chelation blocked the formation of free radicals and, in turn, prevented the production of reactive aldehydes, inflammation and cell death. Further study of LF lotion for large TBSA burns is needed to determine the efficacy of the lotion as an effective alternative treatment for reducing morbidity and scarring long-term.

## Data Availability

If needed, the corresponding author can be contacted to share the data.

## Abbreviations

IL-6: interleukin-6; TNF-α: tumor necrosis factor-alpha; ROS: reactive oxygen species; EDTA: ethylenediaminetetraacetic acid; MSM: methyl sulfonyl methane; LF: Livionex formulation; TUNEL: deoxynucleotidyl transferase biotin-d-UTP nick-end labeling; NIH: National Institutes of Health; TdT: terminal transferase; iNOS: inducible nitric oxide synthase; TBSA: total body surface area.
